# Influence of Flexible and Textile Substrates on Frequency-Selective Surfaces (FSS)

**DOI:** 10.3390/s24051704

**Published:** 2024-03-06

**Authors:** Olga Rac-Rumijowska, Piotr Pokryszka, Tomasz Rybicki, Patrycja Suchorska-Woźniak, Maksymilian Woźniak, Katarzyna Kaczkowska, Iwona Karbownik

**Affiliations:** 1 Faculty of Microsystem Electronics and Photonics, Wrocław University of Science and Technology, Wybrzeże Wyspiańskiego 27, 50-370 Wrocław, Poland; piotr.pokryszka@pwr.edu.pl (P.P.); patrycja.suchorska-wozniak@pwr.edu.pl (P.S.-W.);; 2 Faculty of Electrical, Electronic, Computer and Control Engineering, Technical University of Łódź, Żeromskiego 116, 90-924 Łódź, Poland; tomasz.rybicki@p.lodz.pl (T.R.); ivakabari@gmail.com (I.K.)

**Keywords:** frequency-selective surfaces (FSS), electroconductive textiles, screen printing

## Abstract

Frequency-selective surfaces (FSS) are two-dimensional geometric structures made of conductive materials that selectively transmit or reflect electromagnetic waves. In this paper, flexible FSS made on textile and film substrates is presented and compared to show the effect of the texture associated with the type of substrate on the shielding properties. Three geometries of patterns of squares in the border, inversion of squares in the border, and circles with a border were used, and the patterns were made by the silver paste screen printing technique. Microscopic analysis (SEM and optical) was performed to determine the degree of substrate coverage and the actual geometry of the pattern. The resistance per square of the obtained patterns was about 50 mΩ/□. The shielding properties of FSS were simulated in Comsol Multiphysics 6.2 software and then measured by the antenna method. Selective textile filters were obtained, depending on the pattern used, with one or two modals with a transmission attenuation of about 15 dB. The paper analyzes the effect of the substrate and the screen printing technique used on the shielding properties of the flexible FSS.

## 1. Introduction

Frequency-selective surfaces (FSS) are periodic arrangements of elementary cells in the form of thin dielectric sheets covered with an electrically conductive pattern (quasi 2D) or three-dimensional elements (3D), which exhibit specific frequency selectivity for electromagnetic waves incident on them [1]. The frequency characteristics of such a structure are completely determined by such parameters as the properties of the dielectric substrate (thickness, dielectric permeability), the properties of the conductive material (layer thickness), the surface geometry, the shape and size of a single element (an elemental cell), and the distance between elements on the surface plane. Classical materials that protect against electromagnetic radiation emitted by electrical equipment are designed to operate over the widest possible range of electromagnetic radiation frequencies [2]. FSS materials, on the other hand, are designed to work only at selected frequencies. However, in both cases, the materials must not let electromagnetic radiation pass through, not only by reflection but also by absorption of the electromagnetic field.

Currently, there is a lot of interest in the creation of new and made-from-new materials FSS structures, their research, and their application as frequency selective filters in the microwave to the optical frequency range. In the literature, there are many geometric formulas for typical frequency-selective material structures and the attenuation/absorption/reflection properties of electromagnetic radiation at the corresponding wavelength assigned to them [3]. For example, shapes with dimensions of 5–50 mm operate in the frequency range of 2.5–15 GHz, and the most common patterns are filled or hollow squares, circles or hexagons, crosses, and square spirals [4]. It is also common to use fractals containing multiple repeated elements of different original sizes to achieve multiband FSS, such as Minkowski and Sierpinski fractals [5].

FSS structures are mostly fabricated on rigid, flat, and smooth surfaces made of laminate [6,7], silicon [8], ceramics [9], or glass [10]. Only in recent years has there been interest in flexible FSS materials [11,12,13]. Structures of conductive textiles have attracted attention in recent years, partly due to the growing interest in wearable electronics, i.e., clothing that simultaneously acts as sensors, relays, etc., and also due to the advantages such structures offer: lightweight, flexibility, and softness [14,15]. In the literature, one can find isolated examples of research on textile STFs, as their fabrication is difficult and the resulting structures often do not exhibit the desired parameters due to the lack of symmetry, the texture of the substrate, and the difficulty in accurately reproducing the periodic structure [16]. While on rigid substrates this is not a significant technological problem, making structures on fabrics requires excellent process optimization. In printed processes, where the substrate is subjected to significant stresses, its stability, i.e., the non-deformability of the substrate under different conditions of temperature and stress forces, is of primary importance. Such conditions are usually best suited to thin fabrics with low surface weights, high fill (high thread count), and smooth surfaces. These are usually synthetic multifilament silks such as polyester or polyamide. Due to the elasticity and bending of the fabric, it is necessary to use appropriate techniques to cover them with conductive patterns to obtain FSS of good quality. Ibrahim et al. [17] indicate that weaving conductive threads into the fabric structure during the embroidery process makes it possible to obtain FSS of better quality than when covering the fabric with copper foil patterns, whose mechanical properties are completely different from the textile substrate. For this reason, textile STFs are most often made using the conductive fiber/haft weaving technique [14] or screen printing [18]. Even the use of effective and well-studied techniques for applying conductive layers to substrates requires perfect optimization, which, in addition, will be different depending on the parameters of the textile substrate used: the type and thickness of the yarn from which the substrate is made and the weave of the material. However, the shielding results of textile STFs are always worse than the simulation results due to the texture of the substrate. One way to improve the screening performance is to use an intermediate layer that smooths the fabric before the screen printing process [16] or inkjet printing [19]. Applying structures to fabrics by screen printing requires defining their shapes, and due to the characteristics of fabric substrates (lack of stiffness and surface smoothness), the possible FSS structures are designed primarily for microwave radiation [20]. Thus, fabrics made of multifilament synthetic silks (PET) are needed for screen printing. The preparation of the fabrics must include processes of washing (removal of grease and impurities), drying, and thermal stabilization to ensure the required properties of the fabric, including, above all, its resistance to secondary deformation, smoothness, and uniformity of the surface.

The ability to apply different shapes makes it possible to make narrow-band single [21], dual [22], or even quad-mode [23] FSS filters. With a properly designed resonator geometry, it is possible to obtain a flexible FSS, the efficiency of which will depend only slightly on the polarization of the incident electromagnetic radiation and the change in the shape of the printed sample [22]. This indicates the possibility of using textile systems on clothing or making Meta-Skin FSSs [24], which can be used as a masking element for irregular surfaces.

This paper presents a flexible FSS made by the screen printing technique. Identical structures were made on synthetic substrates—polyethylene terephthalate (PET), but to determine the influence of the texture of the substrate on the quality of the print and the mapping of the pattern geometry, two materials were used: Melinex film with a smooth surface and PET polyester fabric. In addition, the results obtained were related to literature data on identical patterns made on rigid substrates. The results of simulations and tests of the shielding effect of the obtained structures in the microwave range are presented.

## 2. Materials and Methods

### 2.1. Materials

In this work, the following two types of synthetic substrates of polyethylene terephthalate (PET) were used: textiles and foil. The PET textiles were made by WISTIL S.A., a company from Kalisz, Poland. Pre-washed and heat-stabilized (hot air, 20 s, 190 °C) PET silk-type polyester fabric with a surface weight of 80 g/cm^2^ was used for the study. The weight was determined according to PN-ISO 3801:1993—Textile and Fabric Determination of Linear and Surface Mass [25]. Textiles were made in 1/1 plain weave with a thickness of ca. 0.20 mm. The thickness was determined according to EN ISO 5084: 1999—Textile Determination of the Thickness of Textile Products [26]. The average size of macropores in the textiles ranged from 30 to 70 µm.

PET foil, known under the trade name Melinex or Mylar (Selmex company, Plewiska, Poland), is transparent, has a 0.25 mm thickness, and has a surface mass of 349 g/m^2^. Both substrates have high resistance to deformations.

A conductive DuPont screen printing paste from the Intexar series was used, intended for flexible substrates, including textile PE874. Resistivity <50 mΩsq for 25 µm thickness; resistivity after crease (ASTM F1683, 180 deg, 1 cycle, 2 kg) <5%; abrasion resistance (ASTM D3363 Pencil Hardness) 1H; adhesion (tape cross-hatch); and the color is metallic.

### 2.2. Preparation of Frequency-Selective Surfaces

**Figure 1:**
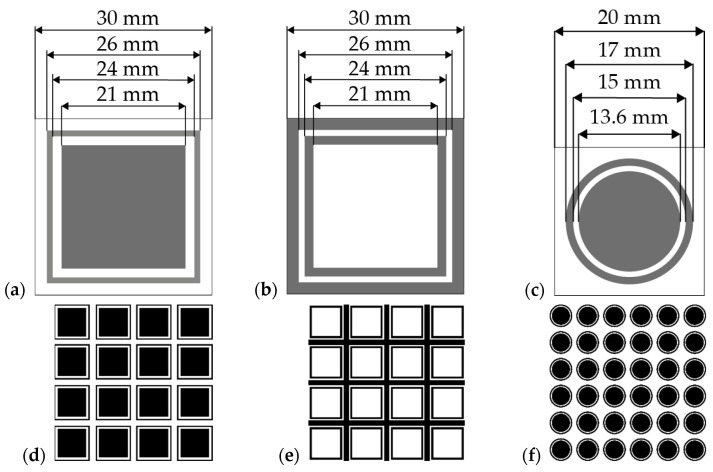
Masks used to make patterns on the screens: (**a**) squares in the border; (**b**) inversion of squares in the border; (**c**) circles with a border; (**d**–**f**) FSS made from elements (**a**–**c**).

The layers were made by screen printing on a semi-automatic screen printer, Uniprint Go3V (PBT WORKS, Lesni, Czech Republic). A 200-mesh steel screen was used to make the prints, on which a photosensitive emulsion with a thickness of 20 μm was applied. In both substrates, i.e., textiles and foil, the layers were applied using PE874 DuPont paste and by printing from one to four layers. After printing, the layers were dried for 15 min at 130 °C in a well-ventilated dryer (Brucker, Peoria, IL, USA). Tree types of prints were made on each substrate, the shape and dimensions of which are shown in Figure 1a–c. The shape of the patterns was taken from the literature in which Shukor et al. [27] presented simulations and execution of FSS on rigid substrates: FR4 and glass. The entire conductive pattern consisted of periodically arranged unit cells (Figure 1d) and consisted of a matrix: 4 × 4 squares in the border (Figure 1a), 4 × 4 inversion squares in the border (Figure 1b), and 6 × 6 squares in the border.

### 2.3. Characterization

The morphology of FSS was determined by the scanning electron microscope Hitachi SU6600 (Tokyo, Japan) and the optical microscope Leica DM4500 B LED (Wetzlar, Germany). To determine the geometry of the printed elements on the textile substrate and film, microscopic images were taken of all the elements at different locations on the sample, and each value was measured a minimum of 10 times using the microscope software LAS X for Leica DM4500 B LED. The study of electrical conductance was a 2-point method. Gold-plated, spring-loaded measurement probes with a diameter of 35 mm and rounded ends were selected for the measurements, which enabled non-destructive measurements and ensured good electrical contact. The probes were placed on a rigid plate, and the whole thing was loaded with two 500 g weights. Before the measurements, the textiles were stored in an atmosphere with a relative humidity of 60% at a temperature of 20 °C for 24 h. The tests were carried out at a constant temperature of 20 °C in an ambient atmosphere with a relative humidity of 30%. DC tests were performed using the Keithley Instruments 610C Solid-State Electrometer device (Cleveland, OH, USA).

The resistance of the printouts was measured ten times and averaged. On this basis, the resistance per square R□ was calculated according to Formula (1).

R□ = R_av_/n (1)

where R□—surface resistance (resistance per square), R_av_—average resistance, and n—number of squares (assumed surface units).

Electromagnetic simulation of FSS structures was performed using the finite element method (FEM) with Comsol Multiphysics software version 5.4 and the RF (Radio Frequency) module. The simulations analyzed the parameters of the scattering matrix S, such as S21, which contains information about the transmission of the electromagnetic wave propagated from the source to the receiver, as well as S11, which carries information about the reflected signal from the tested structure to the source. Simulations were carried out for a substrate made of 0.2 mm thick film and 0.2 mm thick fabric with dielectric permeabilities (εr) of 3.4 [28] and 1.9 [29], respectively. Silver with a resistivity of 50 mΩsq was used as the metallic layer. Geometrical dimensions are given in Figure 1. Electromagnetic simulations were performed for a single elementary cell in the frequency range from 1 to 18 GHz. During the analysis of S-parameters, a virtual matrix of FSS structures of 10 × 10 elements was used to analyze the interaction of an electromagnetic wave with a periodic structure.

**Figure 2:**
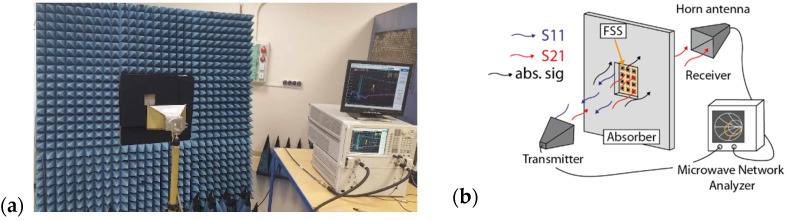
Measurement stand for testing electromagnetic radiation transmission by antenna method: (**a**) photo of the measurement stand; (**b**) schematic diagram with description of the measurement stand.

Shielding properties of FSS were carried out at the Accredited Laboratory for Electromagnetic Compatibility and Electromagnetic Field Measurements (LBEMC), where transmission (S21) measurements were made in the frequency range from 1 to 18 GHz using the antenna method. For this purpose, we used a N5245A vector analyzer from Agilent Technology (Santa Clara, CA, USA) operating in the frequency range of 10 MHz to 50 GHz, an ETS-Lindgren 3115 Horn Antenna (Lindgren, Eura, Finland), enabling measurements from 1 to 18 GHz, and a carbon absorber with a high electromagnetic absorption coefficient of 0.5–40 GHz cat no. MWA-LFA-610 × 610 × 50.8-O (MTC Micro Tech Components, Dillingen an der Donau, Germany). Figure 2 shows (a) a photo and (b) a schematic of the test stand used in the measurement of S-scattering parameters.

## 3. Results and Discussion

### 3.1. Morphology of Frequency-Selective Surfaces

**Figure 3:**
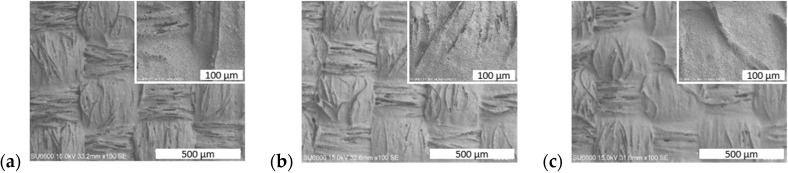
SEM images of PET textile covered by: (**a**) one, (**b**) two, and (**c**) three layers of PE874 paste (inserted image at a higher magnification).

The performance of the fabricated structures having the character of selective band-stop filters largely depends on the quality of the geometric patterns made and the resistivity of the printing, which is closely related to good coverage of the fabric with conductive paste. Images from a scanning electron microscope (SEM) showed that only the printing of three conductive layers ensures complete coverage of the fabric structure (Figure 3). With fewer layers, the pattern produced is discontinuous, and uncovered fibers are still visible on the surface. However, even when the fabric is fully covered, its characteristic weave and surface irregularity are visible, and there is more paste in the recesses than on the convex parts. Moreover, even the continuous layer has a distinctly grainy and heterogeneous structure (Figure 3c, inserted image).

**Figure 4:**
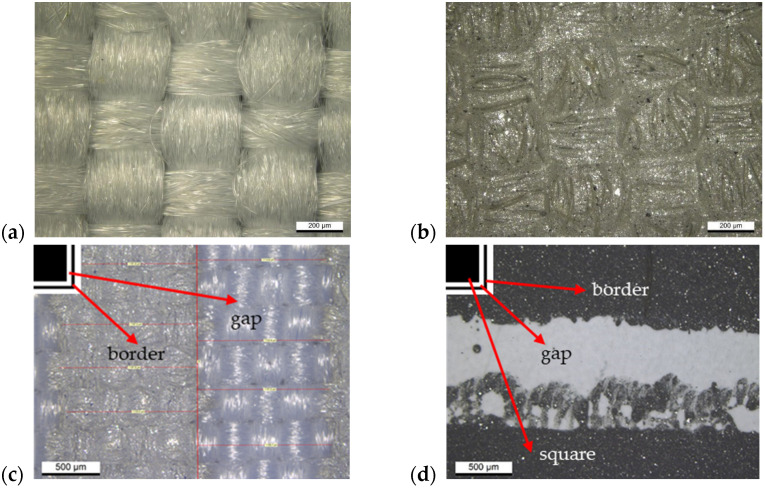
PET textile: (**a**) uncovered and (**b**) covered by three layers. Edges of the squares in the border printed on: (**c**) textiles and (**d**) foils.

The screen printing process is characterized by relatively high printing inaccuracy, which further depends on the type of substrate used. In the case of a textile substrate (Figure 4c), a stepped edge of the print is visible, caused by the placement of the yarn in two directions perpendicular to each other. On the other hand, in the case of the substrate made of foil (Figure 4d), the paste melting at the edge of the print is visible. The blurring seen on the film is a result of the low viscosity of the screen printing paste, which is designed to penetrate well into the textile fabric. For this reason, in many cases, structures made on films had larger deviations from the designed dimensions than structures made on textile substrates, which was confirmed by measurements of all structures (both printed elements and the spaces between them) taken on microscopic images and included in Table 1. The difference in the widths of the individual elements and the gaps between them were in the range of 0.5–17% and 5–23%, respectively, with the gaps between the prints always smaller than designed and the prints smaller or larger (Table 1). These differences are typical values for standard thick-layer technology.

### 3.2. Electrical Resistance

The shielding properties of frequency-selective surfaces depend largely on the resistance of the conductive material used and the quality of the pattern. For this reason, research was carried out to determine the optimal number of layers applied to the textile and foil substrates. The obtained results (Table 2) showed that in the case of fabric, with each layer applied, an increasingly lower resistance per square is obtained, but after applying three layers, it is comparable to the catalog value of 0.050 Ω/□ because then complete coverage occurs of material with conductive paste, as shown by microscopic images (Figure 3 and Figure 4). When using more layers, the resistance per square was lower, but the print quality deteriorated due to the blurring of the pattern. The behavior of the print on Melinex foil is similar. After applying more than three layers, the print was significantly blurred (which, in this case, did not penetrate the fabric), which resulted in a significant increase in resistance. Based on the obtained results, samples of both materials with three layers of silver paste printed on them were selected for testing the shielding properties.

### 3.3. Simulation Results

**Figure 5:**
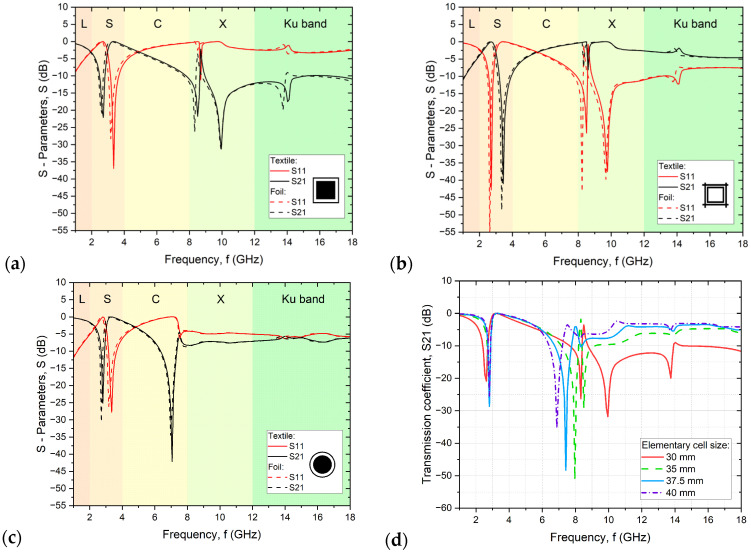
Simulation results for structures printed on foil and textile: (**a**) squares in the border; (**b**) inversion of squares in the border; (**c**) circles with a border (**d**) changes in elementary cell size for structures (**a**,**b**).

The simulations performed show that the use of a two-element pattern consisting of a filled geometric figure (circle or square) in a border of the same shape makes it possible to obtain a dual-mode selective filter, in which the geometric element with a filled conductive layer is responsible for attenuation of radiation at lower frequencies and the ring is responsible for attenuation in the higher frequency range, which is perfectly evident in the case of simulations of the pattern circles with a border (Figure 5c). On the other hand, making patterns that are reciprocal inversions, as in the case of squares in the border (Figure 5a) and inversions of squares in the border (Figure 5b), makes it possible to obtain materials with mutually opposite reflection (S11) and transmission (S21) properties of electromagnetic radiation. However, when designing a multimode filter, it should be borne in mind that a pattern consisting of complex elements, as in the present case, will not behave like the sum of the attenuation of several elements. A structure consisting of squares in the border will not act like the sum of the actions of a structure containing only squares or only borders because both structures interact with each other, and thus the type of interaction depends on the distance between elements. In addition, the distance between elementary cells is also extremely important. When it is too small, it causes the resonance of neighboring rings between each other rather than between a square and a ring. In the case of squares in the border and inversion of squares in the border, the resonance of the interaction of neighboring elements at about 8.5 GHz is visible (Figure 5a), which is not the case in the circles with a border structure. The pattern of circles in a circle is an example of a properly designed structure in which the formation of two narrow bands of attenuation of electromagnetic radiation can be observed.

Therefore, a change in the geometric parameters of structures a and b should be taken into account when designing subsequent designs. Concerning the simulation results (Figure 5d), the distances between elementary cells for structures a and b should be increased to a minimum of 37.5 mm to avoid the negative phenomenon of resonance formation instead of a second attenuation peak. In this publication, as mentioned above, the geometry of resonator elements operating at 3.4 GHz fabricated on rigid substrates was adopted to compare their performance [27].

### 3.4. Shielding Properties

**Figure 6:**
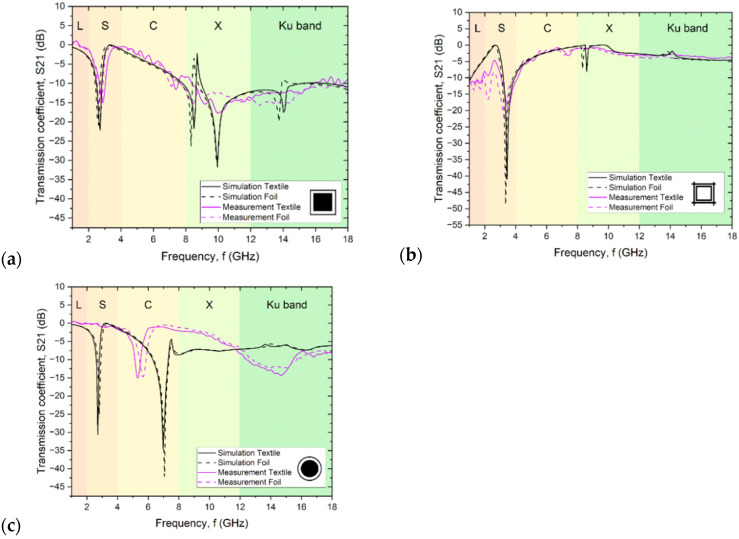
Test and simulation results for structures printed on foil and textile: (**a**) squares in the border; (**b**) inversion of squares in the border; and (**c**) circles with a border.

Measurements of the scattering matrix S using the antenna method made it possible to determine the transmission of the tested structure using parameter S21. It was shown that for all patterns (Figure 6), very similar results were obtained regardless of the substrate used. However, in all cases, there is a slight shift in the characteristics obtained for the print on fabric relative to the print on film, which is mainly due to the difference in the dielectric permeability of the substrate affecting the resonant frequency. The thickness of the substrate in both cases was the same, but the substrates differed in surface texture. The high convergence between the results of the two substrates and the discrepancy between the results obtained and the simulations mean that it is the technique used to make the conductive layer and the material from which it was made that determines the effectiveness of the textile FSS.

The lower radiation attenuation values relative to the simulations are also due to the losses associated with Joule heating (in the conductive layers) and dielectric heating (in the substrate) not included in the simulations [30].

For both squares in the border and their inversion in the lower frequency range, a narrow radiation attenuation peak is evident at 2.4 GHz and 3.2 GHz, respectively, whose presence is associated with a full or hollow square, respectively. These peaks coincide quite well with the values obtained from simulations. Their slight offset from the simulated values is mainly due to the difference in the geometry of the patterns given in simulation and those obtained in reality (Table 1, Figure 4). On the other hand, about half of the weaker attenuation of electromagnetic radiation is related to the quality of prints obtained in the screen-printing process and the discontinuity resulting from the composite composition of the paste, compared to the ideal conductor, i.e., a continuous layer of silver.

The size of the circle width, the border, and the distances between elements were significantly smaller than their counterparts in the squares in the border pattern, which made their implementation much more difficult, and the resulting layer was characterized by quite large imperfections affecting the electrical parameters. Changing the real and imaginary parts of the conductor impedance directly affects the goodness of the filter (Q), that is, the bandwidth fBW, the signal transmission level, and the characteristic frequency (f0). Its change causes a shift in the transmission peak, which is most significant in the case of the pattern of circles with a border (Figure 5c). Disturbances in the mapping of the geometry of the structures have a very significant impact on the shielding efficiency and frequency characteristics of the FSS, which is also confirmed by the study of Turki et al. [31], in which FSS structures were made with intentional pattern disturbances in the form of making incomplete squares, of which the FSS structure consisted.

In the higher-frequency region around 8 GHz in all three structures (Figure 6a–c), the simulations show a second peak associated with the presence of a thin envelope or its inverse, which is absent or much less intense and shifted in the measurement results. These larger deviations of the measured results from the simulated values are the result of a change in the way electromagnetic radiation interacts with the material in the higher frequency range. For the simulations, the parameters of a perfectly smooth layer with equal thickness, constant resistivity, and an electron model of conductivity were assumed. In contrast, screen-printed conductive elements are composite layers consisting of approximately micrometer-sized grains surrounded by polymer. In conductive composite materials, even when the percolation threshold is exceeded, there is always the phenomenon of current tunneling through the thin layer of polymer surrounding the conductive particles. For this reason, even after crossing the percolation threshold, the conductivity of the composite is always 2–4 orders of magnitude lower than that of the filler used [32,33]. As shown by microscopic images (Figure 3), the structure of the layer shows approximately micrometer-sized silver grains, which form a non-uniform, rough layer. In addition, the layer is characterized by high surface heterogeneity due to the shape of the fabric, which is still visible even after three layers were printed (Figure 4b). The inhomogeneity of the layer and the impossibility of 100% reproduction of the assumed shape affect the resistivity of the layer, especially the small and narrow elements such as the borders of squares or circles. There are larger errors in these elements, and they are responsible for the interaction at higher frequencies.

In addition, there is a skin effect in the high-frequency region, causing the current density near the conductor’s surface to be greater than inside the conductor. As a result, there is an increase in the impedance of the conductor, causing an increase in the resulting power losses in the conductor. One of the quantities characterizing the skin effect is the depth of current penetration into the conductor, and in the studied range, it is of the order of several micrometers. In the case of the structures studied, the depth of penetration of the electromagnetic wave is comparable to the roughness and unevenness of the surface due to both the properties of the substrate and the specifics of the screen printing technique. As a result, the electromagnetic wave in such a material is scattered, and there is a change in the conductivity of the material, whose impedance, capacitance, and inductive reactance change value. The result is the practical disappearance of the narrow attenuation peak in the high-frequency range and its replacement by a much wider and less intense peak in the Ku band range.

For peaks near 3 GHz, there is no scattering effect of the high-frequency signal, and in addition, the interacting structures in this range are filled circles or squares, which have better filling and surface coverage and thus lower layer resistance. This makes it possible to produce a narrow-band filter in this frequency range of electromagnetic radiation.

## 4. Conclusions

Textile frequency-selective surfaces utilize textiles as substrates for FSS, offering flexibility, lightweightness, and conformability. This allows them to be used in stealth technology—integrated into clothing or camouflage nets to reduce radar cross-section, making military personnel or equipment less detectable to radar systems; electromagnetic shielding—can be used in clothing or tents to shield against electromagnetic interference (EMI) or electromagnetic radiation (EMR) from sources like cell phones, Wi-Fi routers, or power lines, protecting individuals or sensitive equipment; communication systems—can be employed in wearable antennas for wireless communication devices, such as smartwatches, fitness trackers, or medical monitoring devices, improving signal reception and transmission efficiency; or architectural applications—can be incorporated into building materials, curtains, or window films to control the transmission of specific frequencies of electromagnetic waves, such as blocking harmful UV radiation while allowing visible light to pass through.

Structures containing a square or circular shape in the border are often used in the design of multimode FSSs [3,34,35]. The purpose of this paper was to compare structures fabricated on a textile substrate to analogous structures designed and fabricated on rigid FR4 substrates reported in the literature [27]. In this paper, the reflection (Sl1) and transmission (S21) of the design FSS structure are analyzed. Analyzing the structures with the same design (configuration 1) for the circular loop pattern, two peaks are visible on the transmission spectrum: the first around 1.7 GHz (−35 dB) and the second around 4 GHz, while in the square loop measurements, the first narrow peak is around 1.9 GHz (−26 dB) and the second around 5.5 GHz. In addition, both charts show a general reduction in transmission in the broad spectral range, which occurs already above 3 GHz. This behavior is consistent with our results, in which there are also two peaks on the transmission spectrum and a broad-spectral attenuation of transmission in the high-frequency range is evident. The obtained attenuation for individual peaks in all fabricated structures, both on the textile substrate and on the film, is about 15 dB, or about half that of analogous structures fabricated on the glass-epoxy (FR4) laminate presented in [27]. The simulation results conducted by the authors were consistent with the results available in the literature [27]. However, the actual results obtained from measurements of both foils and fabrics were half as efficient. This is related to the quality of the layers made using screen printing.

The layers made by the screen printing technique are characterized by less continuity than the metallic conductive layers on the laminate, and it is also not possible to reproduce the shape as accurately as in the case of etching the conductive layer in the laminate. While in screen printing it is possible to make structures where the geometric distribution of the elements to each other will be relatively constant, it is more difficult to make elements with assumed geometries and without structure errors within the surface of a single print. Any perturbation of the structure results in the broadening of the peaks, shifting of the peaks, or weakening of the attenuation. In this work, the effect of individual perturbations in the structure of flexible and textile FSSs on their shielding properties is demonstrated. The effects of individual print parameters on the shift of attenuation bands, the reduction or disappearance of electromagnetic radiation attenuation, or the formation of resonances, respectively, are presented.

The structures used in the article and, in particular, squares in the border and the inversion of squares in the border have too small distances between elementary cells. As a result, the resonance between rings in separate elementary cells in the frequency range of 8–10 GHz is visible (Figure 6a,b). Increasing the distance between elementary cells from a value of 30 mm to 37.5 mm for the squares in the border and similarly for the inverted squares should result in the disappearance of the resonance and thus the formation of a two-compartment radiation filter (Figure 5d), which will be the subject of further work by the authors.

## Figures and Tables

**Table 1:** Comparison of design and measured size of printed elements (T—textiles; F—foil).

Sample	Element	Substrate	Design Size [mm]	Measured Size [mm]
squares in the border 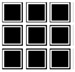	square	T	21	20.0 (+/−0.04)
		F		19.4 (+/−0.18)
	gap	T	1.5	1.16 (+/−0.04)
		F		1.33 (+/−0.06)
	border	T	1	1.17 (+/−0.04)
		F		0.94 (+/−0.44)
	distance	T	4	3.40 (+/−0.04)
		F		3.56 (+/−0.03)
inversion of squares in the border 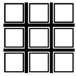	border	T	1.5	1.63 (+/−0.04)
		F		1.70 (+/−0.12)
	gap	T	1	0.92 (+/−0.01)
		F		0.89 (+/−0.34)
	line	T	4	4.2 (+/−0.01)
		F		3.98 (+/−0.05)
circles with a border 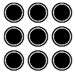	circles	T	1.36	1.30 (+/−0.06)
		F		1.25 (+/−0.06)
	gap	T	7	5.90 (+/−0.06)
		F		6.59 (+/−0.12)
	border	T	10	9.63 (+/−0.03)
		F		9.89 (+/−0.07)
	distance	T	3	2.71 (+/−0.03)
		F		2.73 (+/−0.04)

**Table 2:** Comparison of resistance per square R□ for different numbers of printed layers.

Number of Layers/ Substrate	1 [Ω/□]	2 [Ω/□]	3 [Ω/□]	4 [Ω/□]	5 [Ω/□]
Textile	0.096	0.065	0.057	0.037	0.033
Foil Melinex	0.053	0.050	0.048	0.080	0.090

## Data Availability

Data are contained within the article.
